# Broadband NIRS Cerebral Cytochrome-C-Oxidase Response to Anoxia Before and After Hypoxic-Ischaemic Injury in Piglets

**DOI:** 10.1007/978-3-319-91287-5_24

**Published:** 2018-04-16

**Authors:** Gemma Bale, Ajay Rajaram, Matthew Kewin, Laura Morrison, Alan Bainbridge, Mamadou Diop, Keith St Lawrence, Ilias Tachtsidis

**Affiliations:** 9 Medical Physics and Biomedical Engineering, University College London, London, UK; 10 Medical Biophysics, Western University, and Lawson Health Research Institute, London, Canada; 11 Medical Physics, University College London Hospital, London, UK

## Abstract

Perinatal hypoxic ischaemic (HI) encephalopathy is associated with severe neurodevelopment problems and mortality. This study uses broadband continuous-wave near-infrared spectroscopy (NIRS) to assess the early changes in cerebral oxygenation and metabolism after HI injury in an animal model using controlled anoxia events. Anoxia was induced before and 1 h after various levels of HI injury to assess the metabolic response via the changes in the oxidation state of cytochrome-c-oxidase (oxCCO), a marker of oxidative metabolism. The oxCCO responses to anoxia were classified into five categories: increase, no change, decrease, biphasic and triphasic responses. The most common response (54%) was a biphasic decrease in oxCCO. A change in the classification of the metabolic response to anoxia after HI injury indicated a severe injury, as determined by proton magnetic resonance spectroscopy, with 86% sensitivity. This shows that broadband NIRS can identify disturbances to cerebral metabolism in the first hours after severe HI injury.

## Introduction

Neonatal Hypoxic ischaemic encephalopathy (HIE) affects 1–2 live births per 1000 and is associated with severe neurodevelopmental problems and mortality [1]. After the initial Hypoxic ischaemic (HI) injury, typically the cerebral Metabolism recovers to normal for the first few hours of life but then can deteriorate, leading to a Secondary energy failure (SEF) [2]. HIE is monitored using amplitude-integrated EEG (aEEG) during treatment in the Neonatal intensive care unit (NICU) but the current gold standard assessment of the HIE is proton magnetic resonance spectroscopy (^1^H MRS) [3]; however, this is generally performed towards the end of the first week of life, long after the ‘therapeutic window’ before SEF. A real-time, cotside measurement of cerebral metabolism that can assess the progression of cerebral injury would be helpful in the immediate stages following HI brain injury. An early (within the first 6 h after birth) continuous assessment of brain injury is sought in order to enable prompt treatment and to monitor recovery during the crucial first days following HIE.

Broadband continuous-wave near-infrared spectroscopy (NIRS) can monitor cerebral haemodynamics and metabolism in the brain by measuring the changes in concentration of oxygenated- and deoxygenated-haemoglobin (HbO_2_ and HHb, respectively) and the oxidation state of cytochrome-c-oxidase (oxCCO), which is the terminal electron acceptor in the electron transport chain in mitochondria [4]. Cytochrome-c-oxidase (CCO) contains four redox centres, one of which, copper A, has a broad absorption peak in the near-infrared (NIR) which changes depending on its redox state. In order to detect the small concentration changes in oxCCO, we use broadband (multi-wavelength) NIRS and the UCLn algorithm to accurately resolve spectral changes due to oxCCO without cross-talk from haemoglobin chromophores [4].

We have previously observed that changes in oxCCO during spontaneous desaturation events indicate brain injury severity in neonates with HIEHypoxic ischaemic encephalopathy (HIE) over the first 4 days of life [5]. However, an early biomarker of brain injury (in the first 6 h of life) is necessary to target treatment most effectively. Here we use a piglet model of HI to assess whether broadband NIRS can identify changes in the first hours after injury. The aim of this experiment was to assess metabolic function (via oxCCO) during anoxic challenges before and after HI injury. Further, we wanted to evaluate the classification of injury severity (assessed by ^1^H MRS) based on this response.

## Methods

**Fig. 1:**
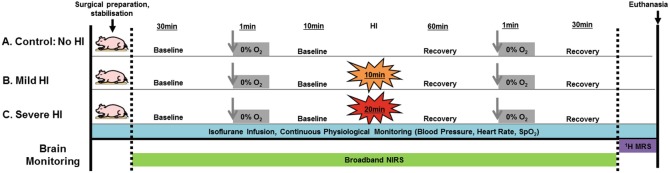
Experimental protocol for the three groups of piglets (control, mild and severe HI)

This study was approved by the Animal Use Subcommittee of the Canadian Council on Animal Care at Western University (London, Ontario). The protocol is shown in Fig. 1. Piglets (2–48 h old) were anaesthetised, tracheotomised, and mechanically ventilated. Incisions were made to place vascular occluders around carotid arteries. HI was induced by inflating the occluders and reducing the fraction of inspired oxygen to 8%; different levels of injury were induced by controlling the duration of HI (control/mild/severe: 0/10/20 min), this was later confirmed when possible with ^1^H MRS [6]. Before and 1 h after HI injury, anoxia (0% FiO_2_ for 1 min) was induced. Broadband NIRS measurements were performed continuously over the left cerebral hemisphere at a sampling frequency of 5 Hz [7]. The changes in chromophore concentrations (oxCCOCytochrome-c-oxidase (oxCCO), HbO_2_ and HHb) were calculated from the measured changes in broadband near-infrared light attenuation using the modified Beer-Lambert law as applied with the UCLn algorithm across 136 wavelengths (771–906 nm) with a fixed differential pathlength factor of 4.39 (preterm head [8]) and 3 cm optode separation.

Systemic data were collected from a SurgiVet monitor (Smiths Medical, USA). Oxygen saturation (SpO_2_) was measured by pulse oximetry on the tail or foot.

Magnetic resonance imaging/spectroscopy was performed after the optical monitoring (~3 h after HI) on a 3 T Biograph mMR scanner (Siemens Healthcare). MR scans included measurements of (Lactate + Threonine)/(NAA + NAAG) (Lac+Thr/tNAA) from the left hemisphere cortex and thalamus with proton (^1^H) MRS (PRESS: TR = 2 s; TE = 135 or 288 ms). MRS data were analysed using Tarquin [9]. The fitted amplitudes of Lac and Thr were combined because they are not resolvable in in vivo spectra [10]; including Thr in the basis set typically yields an improved fit in the region around 1.3 ppm. Lipids and macromolecules were not included in the basis set. Piglets with Lac+Thr/tNAA≥0.3 in either brain structure were determined to have severe HI injury.

Thirty piglets underwent this experiment. Due to technical issues with the data collection (4 piglets) and the fact that 2 piglets died during the insult, data are presented from 24 newborn piglets (14 female): severe HI injury was induced in 13 piglets, mild injury was induced in 6 and there were 5 controls (without HI injury). MRS was successful on piglets 17–32 and confirmed that the longer HI duration induced more severe injury (Lac+Thr/tNAA≥0.3); piglet 25 who experienced HI for 10 min was re-classified to severe as Lac+Thr/tNAA≥0.3. The mean weight was 1.6 ± 0.4 kg and all were within 2–48 h of age.

Data analysis was carried out in MATLAB (Mathworks, USA). The broadband NIRS concentration changes during each anoxic event were selected for the 60 s of 0% FiO_2_ and the proceeding 10 s to include the entire period of cerebral anoxia. The changes in cerebral oxCCOCytochrome-c-oxidase (oxCCO) were plotted against the changes in cerebral HbO_2_ to assess the metabolic response to oxygenation decrease in the brain. These were classified into five different metabolic responses for each event by observation: increase, no change, linear decrease, biphasic decrease, and triphasic decrease (see Fig. 3 for examples). The sensitivity and specificity of the ability of the different metabolic responses to predict a severe injury was calculated across all groups.

## Results

**Fig. 2:**
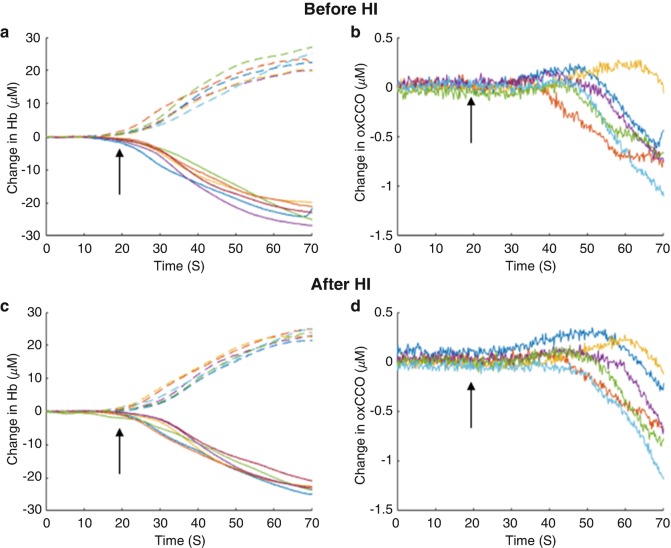
Examples (piglets 9–16) of anoxic changes in chromophore concentrations before (**a**, **b**) and after (**c**, **d**) HI: (**a**, **c**) HbO_2_ (solid) and HHb (dashed), (**b**, **d**) oxCCO. FiO_2_ was switched to 0% at 0 s and returned to 21% at 60 s; arrows indicate the start of cerebral anoxia

**Fig. 3:**
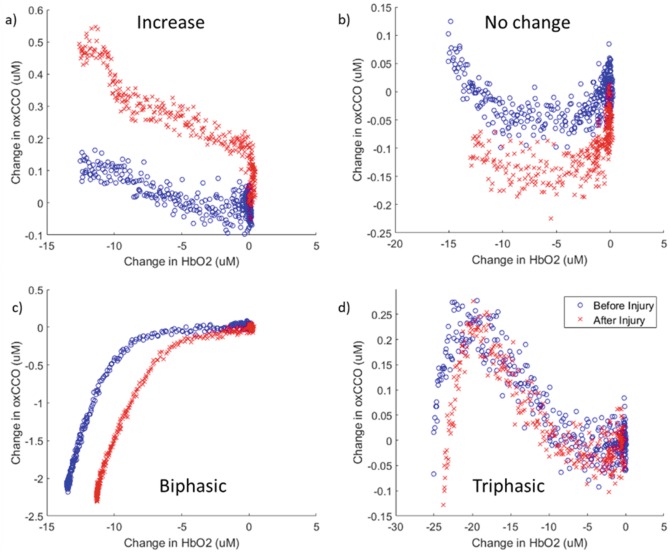
Examples of oxCCO responses to anoxia, before (blue circles) and after HI (red crosses). (**a**) Piglet 07 (control) shows an increase. (**b**) Piglet 10 (severe HI) showing no change. (**c**) Piglet 08 (control) shows a biphasic change. (**d**) Piglet 13 (severe HI) shows a triphasic change

**Fig. 4:**
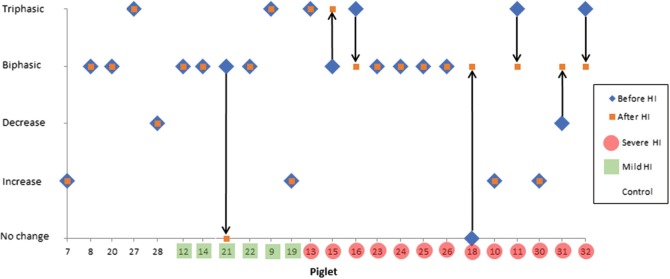
Classification of response of oxCCOCytochrome-c-oxidase (oxCCO) to decreased HbO_2_ during anoxia. Note that most (86%) of oxCCO responses which changed after HI were in piglets that experienced severe HI

Figure 2 shows examples of the response to anoxia before and after (mild or severe) HI. A decrease in HbO_2_ and increase in HHb is observed in all cases, but the changes in oxCCO were different and could be split into five categories; in 17% of events an increase in oxCCO was observed, decrease in oxCCO was seen in 6% of events, no change in oxCCO was observed in 4% of events, in 19% of events a triphasic change in oxCCO was seen; but the most common response was a biphasic decrease in oxCCO which occurred in 54% of events. Examples of the changes in oxCCO with HbO_2_ are shown in Fig. 3. The magnitude of change was not related to the injury level for any of the chromophores. Figure 4 shows a summary of the oxCCO responses to anoxia, before and after HI. There was no link between the classification of oxCCO response and the level of HI injury. However, almost all of the piglets in which the oxCCO response changed from before to after HI injury had severe HI injury (86% sensitivity, 46% specificity).

## Discussion

We observed five different cerebral metabolic responses to anoxia, despite similar cerebral haemodynamic responses across all piglets. This metabolic heterogeneity has been observed before in piglets during different levels of hypoxia; Tsuji et al. observed both increases and decreases in oxCCO [11]. The most common response was a biphasic reduction in oxCCO (example in Fig. 3b) which has previously been reported in piglets [12]. The interpretation of the ‘biphasic’ responses is that at high oxygen tensions, oxCCO is independent of oxygenation; as the cellular oxygen tension reduces to critical levels, the oxidation state of CCO becomes more reduced [12]. An increase in oxCCO, observed initially in the ‘triphasic’ responses and in the ‘increase’ responses, suggests that the mitochondria increase oxidative Metabolism under some conditions of stress [11].

Interestingly, many different responses were observed in the control group and before HI (i.e. in healthy, anaesthetised piglets). Nonetheless, the oxCCO response was consistent and repeatable per piglet in the control group and the majority (84%) of the mild HI injury groups. However, a change in the oxCCO response to anoxia after HI injury indicated severe injury with high sensitivity. This suggests that severe HI injury disrupts the ‘normal’ metabolic function and augments the metabolic response to anoxia. Mitochondrial dysfunction is therefore apparent early (1 h) after HI injury, but only in the most severe cases of injury. After severe HI the mitochondria may not have fully recovered from the insult or may be experiencing the beginnings of secondary energy failure. There were heterogenous responses to anoxia and seven of the severe injury piglets did not change their oxCCO response to anoxia. This could be due to several confounding factors that affect the metabolic response to anoxia, such as the heterogeneity of the piglets (gender, birth weight, etc.) or underlying physiological changes during the anoxia. We observed that the blood pressure response (data not presented) was heterogenous, with some piglets having very low drops in blood pressure during anoxia. This is a limitation of our study and the impact of the systemic physiology needs to be investigated further.

In conclusion, these results show that broadband NIRS is able to separate severe HI from mild HI very early after the injury by using anoxic stimuli and observing the response before and after the injury. We can identify mitochondrial dysfunction 1 h after HI in animal models; this is promising for the use of broadband NIRS early in the clinic, however a more appropriate stimulus needs to be developed. The ultimate goal is to use broadband NIRS to identify the worst cases of Near-infrared spectroscopy Hypoxic ischaemic encephalopathy (HIE) immediately after birth and propose treatment accordingly.

## References

[1] Finer NN, Robertson CM, Richards RT, Pinnell LE, Peters KL (1981). Hypoxic-ischemic encephalopathy in term neonates: perinatal factors and outcome. J Pediatr.

[2] Lorek A, Takei Y, Cady E, Wyatt J (1994). Delayed (‘Secondary’) cerebral energy failure after acute hypoxia-ischemia in the newborn piglet; continuous 48-hour studies by phosphorus magnetic resonance spectroscopy. Pediatr Res.

[3] Thayyil S, Chandrasekaran M, Taylor A, Bainbridge A, Cady EB, Chong WKK, Murad S, Omar RZ, Robertson NJ (2010). Cerebral magnetic resonance biomarkers in neonatal encephalopathy: a meta-analysis. Pediatrics.

[4] Bale G, Elwell CE, Tachtsidis I (2016). J Biomed Opt.

[5] Bale G, Mitra S, de Roever I, Sokolska M, Price D, Bainbridge A, Uria-Avellanal C, Kendall GS, Meek J, Robertson NJ, Tachtsidis I (in press) Oxygen dependency of mitochondrial metabolism indicates outcome of newborn brain injury. J Cereb Blood Flow Metab 10.1177/0271678X18777928 PMC6775592 29775114

[6] Tichauer KM, Elliott JT, Hadway JA, Lee DS, Lee T-Y, Lawrence KS (2010). Using near-infrared spectroscopy to measure cerebral metabolic rate of oxygen under multiple levels of arterial oxygenation in piglets. J Appl Physiol.

[7] Diop M, Kishimoto J, Toronov V, Lee DSC, Lawrence KS (2015) Development of a combined broadband near-infrared and diffusion correlation system for monitoring cerebral blood flow and oxidative metabolism in preterm infants. Biomed Opt Express 6(10):3907–3918 10.1364/BOE.6.003907 PMC4605050 26504641

[8] Wyatt JS, Cope M, Delpy DTD, van der Zee P, Arridge S, Edwards AD, Reynolds EOR (1990). Measurement of optical path length for cerebral near-infrared spectroscopy in newborn infants. Dev Neurosci.

[9] Wilson M, Reynolds G, Kauppinen RA, Arvanitis TN, Peet AC (2011). A constrained least-squares approach to the automated quantitation of in vivo (1)H magnetic resonance spectroscopy data. Magn Reson Med.

[10] Kreis R, Hofmann L, Kuhlmann B, Boesch C, Bossi E, Hüppi PS (2002). Brain metabolite composition during early human brain development as measured by quantitative in vivo 1H magnetic resonance spectroscopy. Magn Reson Med.

[11] Tsuji M, Naruse H, Volpe J, Holtzman D (1995). Reduction of cytochrome aa3 measured by near-infrared spectroscopy predicts cerebral energy loss in hypoxic piglets. Pediatr Res.

[12] Springett R, Newman J, Cope M, Delpy DT (2000). Oxygen dependency and precision of cytochrome oxidase signal from full spectral NIRS of the piglet brain. Am J Physiol Heart Circ Physiol.

